# Comparison of the Effects of Mesenchymal Stem Cells with Their Extracellular Vesicles on the Treatment of Kidney Damage Induced by Chronic Renal Artery Stenosis

**DOI:** 10.1155/2020/8814574

**Published:** 2020-10-08

**Authors:** Crysthiane Saveriano Rubiao Andre Ishiy, Milene Subtil Ormanji, Edgar Maquigussa, Rosemara Silva Ribeiro, Antonio da Silva Novaes, Mirian Aparecida Boim

**Affiliations:** Renal Division, Department of Medicine-Federal University of São Paulo, São Paulo, Brazil

## Abstract

**Background:**

Chronic renal artery stenosis is considered one of the most common causes of renovascular hypertension (RH). Chronic hypoxia can lead to irreversible damage to renal tissue and to a progressive deterioration of renal function. We have previously shown that bone marrow-derived mesenchymal stem cells (BMSCs) improved renal parenchyma and function in a model of RH (2 kidneys, 1 clip model (2K-1C) in rats. Microvesicles (MVs) and exosomes (EXs) released by MSCs have been shown to induce effects similar to those induced by whole cells but with fewer side effects. In this study, we compared the effects of adipose-derived MSCs (ASCs) with those of the MVs and EXs released by ASCs on tissue inflammation and renal function in 2 K-1C rats.

**Results:**

Flow cytometry analysis showed that even after 15 days, ASCs were still detected in both kidneys. The expression of a stem cell homing marker (SDF1-*α*) was increased in ASC-treated animals in both the stenotic and contralateral kidneys. Interestingly, SDF1-*α* expression was also increased in MV- and EX-treated animals. A hypoxia marker (HIF1-*α*) was upregulated in the stenotic kidney, and treatments with ASCs, MVs, and EXs were effective in reducing the expression of this marker. Stenotic animals showed a progressive increase in systolic blood pressure (SBP), while animals treated with ASCs, MVs, and EXs showed a stabilization of SBP, and this stabilization was similar among the different treatments. Stenotic animals developed significant proteinuria, which was reduced by ASCs and MVs but not by EXs. The increased expression of Col I and TGF*β* in both kidneys was reduced by all the treatments, and these treatments also effectively increased the expression of the anti-inflammatory cytokine IL-10 in both kidneys; however, only ASCs were able to reduce the overexpression of the proinflammatory cytokine IL-1*β* in both kidneys of 2K-1C animals.

**Conclusion:**

The results of this study demonstrated that the EVs released by ASCs produced beneficial results but with lower efficacy than whole cells. ASCs produced stronger effects in this model of renal chronic hypoxia, and the use of EVs instead of whole cells should be evaluated depending on the parameter to be corrected.

## 1. Introduction

Chronic renal artery stenosis is considered to be one of the most common causes of renovascular hypertension (RH) [1]. In addition to hypertension, renal artery stenosis causes chronic hypoxia in the kidney and can lead to irreversible renal tissue injury and progressive deterioration of renal function. The prevalence of RH is estimated to be 2% of the general population of hypertensive patients, and RH has become an important cause of end-stage renal disease, especially in elderly patients [1–4]. Chronic hypoxia is characterized by microvascular rarefaction leading to irreversible damage to the renal parenchyma. In fact, clinical studies have reported that even after resolution of stenosis through angioplasty, the recovery of renal function is limited, and patients often progress to end-stage chronic renal disease [5–8]. In addition, during unilateral renal stenosis, the contralateral kidney is constantly exposed to high blood pressure and exhibits structural and functional features of a hypertensive kidney.

Due to the limited treatment options and the reduced angiogenic and regenerative abilities of the kidney, cellular therapy appears to be a potential therapeutic strategy. Recent studies from our group have shown that mesenchymal stem cells (MSCs) obtained from bone marrow (BMSCs) produced significant beneficial effects in the experimental model of RH proposed by Goldblatt et al. (2K-1C) [9]. Through paracrine effects, BMSCs stimulated angiogenesis, improving microvascular rarefaction, increasing immunomodulation, and resulting in reduced fibrogenesis and proteinuria. All these effects contributed to the prevention of the progressive increase in blood pressure [10]. BMSC treatment also improved the morphology and attenuated the expression of proinflammatory cytokines in the contralateral kidney [11]. Although MSCs have produced promising results in the improvement of kidney architecture and function, the side effects and potential complications observed after cell transplantation, including tumor formation, thrombosis, and capillary obstruction, must be considered [12].

Extracellular vesicles (EVs) produced and released by different cell types, including MSCs, may be an alternative to whole cells in many strategies of cellular therapy. EVs contain many active molecules, such as DNA fragments, mRNAs, microRNAs, and proteins, that play important roles in cellular communication [13, 14]. EVs may influence target cell behavior by transferring their intravesicular content. Microvesicles (MVs) and exosomes (EXs) consist of heterogeneous populations of EVs that differ according to their biogenesis, size, and sedimentation rate [15]. Recent evidence indicates that MSC-conditioned medium containing EVs exerts therapeutic effects similar to those of whole MSCs [16–18]. EVs have been demonstrated to induce renoprotective effects during ischemic acute kidney injury by restoring morphology, promoting angiogenesis and cell proliferation, reducing fibrosis, and restoring renal function [14, 19, 20]. MVs and EXs may have distinct effects, since they can carry different contents in terms of species and/or amounts, and as recently demonstrated by Bruno et al. [21] in an acute kidney injury model, distinct MSC-derived EV populations have different regenerative effects.

On the other hand, the role of distinct populations of EVs in chronic renal ischemia models has been less explored. Therefore, the aim of this study was to investigate and compare the effects of MSCs obtained from adipose tissue with those of their MVs and EXs in a model of chronic renal ischemia induced by partial clamping of the left renal artery (2K-1C model).

## 2. Materials and Methods

Male Wistar rats (150–180 g) were purchased from the animal facility (CEDEME) of the Federal University of São Paulo. Animals were housed in cages in groups, were acclimated to a room temperature of 23°C with a 12-h light/dark cycle, and were given free access to rat chow and tap water. All the experimental procedures were approved by the Ethics in Research Committee of the Federal University of São Paulo (CEUA-6972080514).

### 2.1. Induction of Renal Artery Stenosis (2K-1C Model)

Animals were anesthetized with intraperitoneal (IP) injections of 40 and 20 mg/kg ketamine and xylazine, respectively (Vetbrands, SP, Brazil). After a peritoneal incision, the left renal artery was partially obstructed with a 0.2 mm silver clip (Sigma-Aldrich, MO, USA) as previously described [9, 10]. The control animals (sham) were subjected to the same surgical procedure but without renal artery occlusion. Animals were observed for six weeks.

### 2.2. Systolic Blood Pressure

Systolic blood pressure (SBP) was measured weekly by plethysmography (PowerLab-ADInstruments, CH, Australia). Each animal was maintained in a heating box for 10 minutes to promote the vasodilatation of the caudal vein and to facilitate measurement of the SBP with a tail-cuff sensor that was connected to a computerized system (LabChart-ADInstruments, CH, Australia) with specific integration software (PowerLab-ADInstruments).

### 2.3. Adipocyte Mesenchymal Stem Cell (ASC) Isolation and Characterization

White adipose tissue was removed from the gonadal region of 6-week-old rats under sterile conditions and washed with PBS (phosphate-buffered saline). The tissue was digested enzymatically at 37°C for 40 minutes with constant stirring in 2 ml of low-glucose DMEM containing 0.1% collagenase type 1A (Sigma-Aldrich, MO, USA) and 20 mg of bovine serum albumin (Sigma-Aldrich). The enzymatic activity was quenched with the same volume of fetal bovine serum (FBS, Thermo Fisher Scientific, MA, USA); the digested adipose tissue was centrifuged at 400 g for 15 minutes and resuspended in low-glucose DMEM supplemented with 10% FBS and 1% penicillin/streptomycin (PS). Subsequently, the resuspended tissue was placed in 10 mm diameter plastic dishes and incubated in an incubator at 37° C with 5% CO_2_ for 2 hours. After this period, all the medium containing nonadherent cells was discarded. Fresh culture medium with the same concentrations of FBS and antibiotics was then added. During the three days after extraction, the culture medium was changed every 24 hours to gradually remove the red blood cells and cell debris from the extracted cells.

Immunophenotype assays were performed with antibodies against CD73, CD90, CD29, CD105, CD31, CD34, and CD45 (Becton Dickinson, NJ, USA), and the samples were analyzed by flow cytometry (FACS Canto, Becton Dickson, NJ, USA). ASC multipotentiality was evaluated by adipogenic and osteogenic differentiation. To induce differentiation, 1 × 10^4^ cells/cm^2^ were placed in each well of a 6-well culture dish in triplicate. The conventional culture medium (low-glucose DMEM+10% FBS) was replaced with adipogenic or osteogenic medium (StemPro Adipogenesis Differentiation Kit and StemPro® Osteogenesis Differentiation Kit, respectively; Invitrogen, MA, USA) for 14-21 days, and the medium was changed every 3 days. Differentiation was confirmed with specific oil red staining for adipocytes and Alizarin S staining for osteoblasts. Osteogenic differentiation was demonstrated by the accumulation of calcium in the extracellular matrix, as shown by Alizarin Red staining, on days 14 and 21 of differentiation. Adipogenic differentiation was confirmed by fixing the cells for 1 hour with 4% paraformaldehyde and staining with 0.9% Oil red. After a 5-minute incubation period, the cells were washed with deionized water, and the lipid vacuoles were visualized by orange staining under an inverted microscope (Nikon, TKY, Japan).

### 2.4. Extracellular Vesicle (EV) Isolation and Characterization

EVs were purified from the ASC culture medium and separated according to size into fractions enriched with microvesicles (MVs) or exosomes (EXs) by ultracentrifugation. The ASCs were cultured in 75 cm^2^ flasks containing low-glucose DMEM supplemented with 10% FBS and 1% PS until they reached 90% confluency. After this period, the culture medium was changed to low-glucose DMEM with 1% PS but without FBS. The cell supernatant was collected after 72 hours and centrifuged at 3,000 g for 20 minutes to remove cellular debris and apoptotic bodies. This step was followed by ultracentrifugation (Hitachi, TKY, Japan) at 10,000 g for 30 minutes to obtain microvesicles and then at 100,000 g for 2 hours to obtain exosomes [22]. The vesicles were identified by Nanosight following the manufacturer's protocol, and the expression of MMP2 (MV) and CD63 (EX) (Abcam, MA, USA) was analyzed by Western blotting.

### 2.5. Experimental Protocol

The rats were divided into five groups: sham (*n* = 7), stenotic (*n* = 7), stenotic+ASC (ASC, *n* = 7), stenotic+MV (MV, *n* = 7), and stenotic+EX (EX, *n* = 7). The ASCs, MVs, and EXs were infused through the tail vein at the 3^rd^ and 5^th^ weeks after clamping. The ASCs were injected at a density of 2 × 10^5^ cells [23] diluted in 200 *μ*l of PBS [10]. The EV-treated groups received 100 *μ*g of MVs or EXs [14, 20] diluted in 200 *μ*l of PBS. Animals were euthanized 6 weeks after clipping by the intraperitoneal injection of an anesthetic overdose of ketamine (160 mg/kg) and xylazine (80 mg/kg).

### 2.6. Cell Labeling for Tracking Assay

To assess the migration and retention time of the ASCs in the tissues, the cells were incubated with Qtracker 585 nm (Invitrogen, CA, USA) following the protocol recommended by the manufacturer. The cells were administered to an additional group of stenotic rats six weeks after renal artery clamping, and then, four groups were established according to the period after ASC administration: 24 hours (*n* = 3), 48 hours (*n* = 3), 72 hours (*n* = 3), and 15 days (*n* = 3). Animals that did not receive treatment were used as controls. Fragments of the left (stenotic) and right (contralateral) kidneys, heart, and lungs were homogenized through a 70 *μ*M cell filter (Becton Dickinson, Franklin Lakes, NJ, USA), and the cells were washed twice in ice-cold PBS and analyzed by flow cytometry (FACSCanto, Becton Dickinson, NJ, USA).

### 2.7. Assessment of Renal Function

At the end of six weeks, animals from all the experimental groups were housed in metabolic cages for 24 h urine collection. Then, the animals were anesthetized with ketamine and xylazine, and aortic blood samples were collected. The plasma and urinary concentrations of sodium, potassium (9180 Electrolyte Analyzer, Roche Diagnostics, Indianapolis, USA), and creatinine (Labtest Diagnostics, Lagoa Santa, Brazil) and the urinary excretion of protein (Labtest Diagnostics, Lagoa Santa, Brazil) were determined. Both kidneys were harvested, the cortex and medulla were quickly separated on ice, and the fragments were destined for gene expression analysis.

### 2.8. Gene Expression Analysis (qPCR)

Total RNA was obtained from the cortex and medulla of both kidneys by the phenol and guanidine isothiocyanate-cesium chloride method using the TRIzol kit (Ambion, CA, USA), according to the manufacturer's instructions. Two micrograms of total RNA was treated with DNase (Promega, WI, USA) to avoid genomic DNA contamination and reverse-transcribed into cDNA by the addition of a mixture containing 0.5 mg/ml oligo(dT) (Life Technologies, CA, USA), 10 mM DL-dithiothreitol (Life Technologies, CA, USA), 0.5 mM deoxynucleoside triphosphates (Life Technologies), and 200 units of reverse transcriptase enzyme (SuperScript RT II; Life Technologies). The mRNA expression levels were estimated using qPCR (QuantStudio 7; Applied Biosystems, CA, USA) by the TaqMan or SYBR Green qPCR methods. The specific TaqMan Assay primer sets were as follows: IL-10 (Rn00563409_m1), IL-1*β* (Rn00580432_m1), *β*-actin (Rn00667869_m1), and HIF-1*α* (Rn1472831_m1) (Life Technologies, NY, USA). The following forward and reverse primers used for the SYBR green assays were as follows (forward and reverse, respectively): *β*-actin (5′ cctctatgccaacacagtgc 3′ and 5′ acatct-gctggaaggtggac 3′), TGF*β* (5′ tgacgtcactggagttgtacgg 3′ and 5′ aactattgcttcagctccacagaga 3′), and SDF-1*α* (5′ gagccatgtcgccagagccaac 3′ and 5′ cacacctctcacatcttgagcctct 3′). The comparative CT method (*ΔΔ*CT) was employed to estimate the gene expression, and the relative mRNA levels were calculated as 2^-*ΔΔ*CT^. The mRNA expression levels were normalized to *β*-actin expression, which was used as an endogenous control.

### 2.9. Western Blot Analysis

Total protein was extracted from microvesicles and exosomes in ice-cold buffer [50 mM TRIS (Sigma-Aldrich, MO, USA), 150 mM NaCl (Labsynth, SP, Brazil), 1.0% nonidet-P-40 (Bio-Rad Laboratories, CA, USA), 0.5% sodium deoxycholate (Sigma-Aldrich), and 0.1% SDS (pH 8.0; Sigma-Aldrich) containing protease inhibitors (AEBSF, aprotinin, bestatin, E-64, leupeptin, pepstatin A; Protease Inhibitor Cocktail; Sigma-Aldrich)] and quantified using a modified Lowry method (Bio-Rad, HH, UK). The protein samples (50 *μ*g) were separated according to size by 12% SDS-PAGE and electroblotted onto nitrocellulose membranes (GE Life Sciences, LC, UK). The membrane blots were probed with primary antibodies overnight at 4°C and with HRP-conjugated secondary antibodies for 1 h at 4°C. The primary antibodies used were as follows: anti-MMP2 diluted at 1 : 100 (Abcam, MA, USA) for the MVs and anti-CD63 diluted at 1 : 100 (Abcam, CBG, UK) for the EXs. Next, the membranes were incubated with HRP-conjugated secondary antibodies (GE Life Sciences). The protein bands were visualized using the Immobilon Western HRP substrate (Millipore, MO, USA). The obtained bands were quantified using Uvitec analysis software (Uvitec Limited, CBG, UK).

### 2.10. Statistical Analysis

The results are represented as the mean ± standard deviation. The data were analyzed using one-way ANOVA followed by the Tukey or Newman Keuls posttests when appropriate. The blood pressure data were analyzed using two-way ANOVA followed by the Bonferroni posttest. Statistical significance was defined as *p* < 0.05. The data were analyzed statistically using GraphPad Prism 5 software (GraphPad Software, CA, USA).

## 3. Results

### 3.1. Characterization of ASCs and MVs

ASCs were characterized according to their capacity for osteogenic and adipogenic differentiation. For the control, the ASCs were maintained in standard media, and no differentiation was observed (Supplementary Figure 1A). To evaluate osteogenic differentiation, the ASCs were incubated with osteogenic differentiation media for 21 days and then subjected to the Alizarin Red staining protocol, which stained the calcium deposits (Supplementary Figure 1B). To evaluate adipogenic differentiation, the ASCs were incubated with differentiation media and exhibited a phenotypic change similar to adipocytes after 14 days of culture. After staining the cells with Oil Red to visualize the cytoplasmic accumulation of lipids, red staining was observed, as shown in Supplementary Figure 1C. After the third passage, the ASCs exhibited a negative staining pattern for hematopoietic markers (CD34 and CD45) and the endothelial marker (CD31). The ASCs exhibited a positive staining pattern for the adhesion marker (CD29) and mesenchymal markers (CD105, CD90, CD73, and CD44) (Supplementary Figure 1D).

The ASCs were cultured without the addition of FBS to obtain conditioned medium. Three time points (24 h, 48 h, and 72 h) were established for cell survival and viability analysis. As shown in Supplementary Figure 2A-B, there was no significant difference in the survival rate and cell viability at 24 h, 48 h, and 72 h. These data demonstrate that ASC culture without FBS does not affect the cell viability and apoptosis of these cells within 72 h. Due to the higher percentage of EVs/ml in the culture media at the 72 h time point (data not shown), we chose this time point for the isolation of extracellular vesicles.

The characterization of the extracellular vesicles was first performed through the analysis of the concentration and size of the nanoparticles by Nanosight. The microvesicles exhibited variable and larger sizes (115 ± 1 vs. 57 ± 36; *p* < 0.05) compared to the exosomes (Supplementary Figure 2C), demonstrating that ASCs release vesicles that are compatible with both microvesicles and exosomes in terms of size. Fifty micrograms of protein from the MVs and EXs was subjected to Western blotting analysis using antibodies against MMP2 as a marker of microvesicles and CD63 as a marker of exosomes; the results are shown in Supplementary Figure 2D. These data indicate that these proteins can be used to confirm the presence of each type of EV derived from ASCs.

### 3.2. ASC Tracking and Distribution

To analyze the retention time of the ASCs in the different tissues, Qtracker®-labeled ASCs were injected 6 weeks after the induction of renal artery stenosis, and 4 groups were established according to the time point after the administration of the ASCs (24 hours, 48 hours, 72 hours, and 15 days). These groups were analyzed by flow cytometry (Supplementary Figure 3). The highest percentage of cells in the tissues was observed at 48 hours, and these cells were mainly located the left kidney (28%) and right kidney (28%). The cells were also found in the heart (0.5%) and lung (4%), and after a few days, the presence of ASCs in the lungs (0.05%) decreased considerably, while the percentage of cells in the left kidney (15%) and right kidney (19%) remained elevated, even 15 days after administration. Other animal groups received 2 injections of ASCs at the 3^rd^ and 5^th^ weeks after clamping. These groups also showed retention of these cells in the right kidney (16%) and the left kidney (22%).

### 3.3. Effect of Treatments on SBP

**Figure 1:**
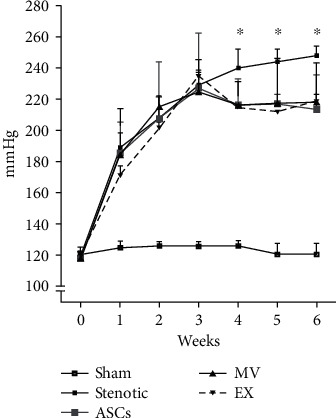
Systolic blood pressure. Values were recorded weekly by plethysmography for six weeks. Groups: sham (*n* = 10), stenotic (*n* = 7), stenotic+ASC (*n* = 7), stenotic+MV (*n* = 7), and stenotic+EX (*n* = 7). SBP is presented as the mean ± SD. ^∗^*p* < 0.05 (two-way ANOVA followed by Bonferroni posttest).

The basal SBP values were similar among the groups (Figure 1), demonstrating that the conditions of the animals used in the experiments were similar prior to the procedure. After renal artery clamping, all stenotic animals exhibited a progressive increase in SBP until the 3^rd^ week. At that time point, the treated groups (ASCs, MVs, and EXs) showed a slight reduction in SBP, which was followed by a stabilization of SBP in the 4^th^ week and no additional increases until the 6^th^ week. These results were different from those observed in stenotic nontreated animals.

### 3.4. Effect of Chronic Renal Artery Stenosis on Body, Kidney, and Heart Weights

**Figure 2:**
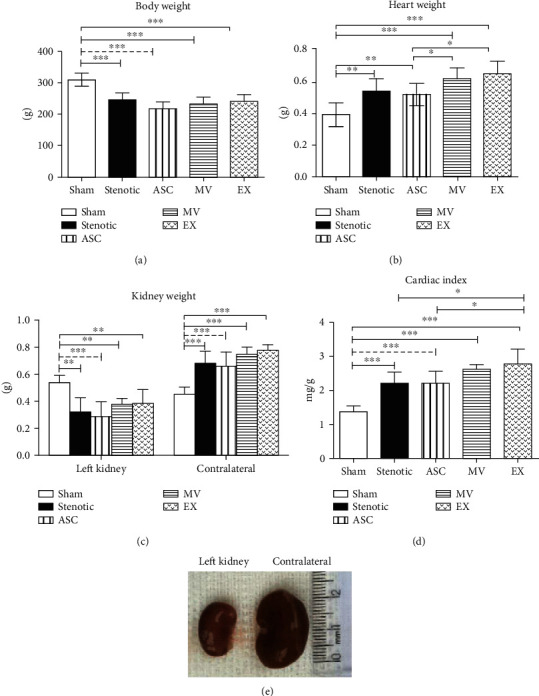
Body weight (a), heart weight (b), kidney weight (c), and cardiac index (d). Groups: sham (*n* = 6), stenotic (*n* = 6), stenotic+ASC (*n* = 6), stenotic+MV (*n* = 6), and stenotic+EX (*n* = 6). Representative images from the stenotic (left) kidney and contralateral kidney after occlusion (e). Data are presented as the mean ± SD. ^∗^*p* < 0.05, ^∗∗^*p* < 0.01, and ^∗∗∗^*p* < 0.001 (one-way ANOVA followed by the Newman-Keuls posttest).

Hypertensive animals showed lower body weight (BW) than sham animals, and the change in BW was not affected by any of the treatments (Figure 2(a)). In contrast to BW, stenotic animals showed a significant increase in heart weight (Figure 2(b)) compared to sham animals, resulting in an increase in the cardiac index (Figure 2(d)). Cardiac hypertrophy was not reversed by any of the treatments (Figure 2(b)). There was a significant reduction in the kidney weight of stenotic animals, whereas the contralateral kidney weight of stenotic animals was significantly higher than that of sham animals. Similar to the heart weight, none of the treatments modified the kidney weight (Figures 2(c) and 2(e)).

### 3.5. Renal Function Parameters

**Figure 3:**
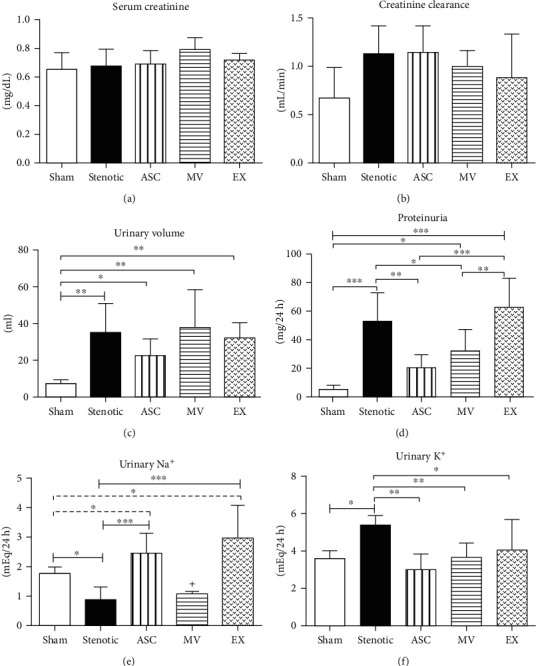
Renal function parameters. Serum creatinine (a), creatinine clearance (b), urinary volume (c), proteinuria (d), urinary sodium excretion (e), and urinary potassium excretion (f). Groups: sham (*n* = 6), stenotic (*n* = 6), stenotic+ASC (*n* = 6), stenotic+MV (*n* = 6), and stenotic+EX (*n* = 6). Data are presented as the mean ± SD. ^∗^*p* < 0.05, ^∗∗^*p* < 0.01, ^∗∗∗^*p* < 0.001, and ^+^*p* < 0.001 (one-way ANOVA followed by the Newman-Keuls posttest).

There was no significant change in the serum creatinine levels (Figure 3(a)) or in the estimated GFR (creatinine clearance, Figure 3(b)) among the groups. In contrast, significant increases in the urinary volume were observed in stenotic animals (Figure 3(c)). Polyuria is a typical manifestation of this model, and none of the treatments modified the urinary volume. Despite the absence of detectable changes in serum creatinine, signs of renal dysfunction could be observed in stenotic animals, such as the increase in proteinuria (Figure 3(d)). Interestingly, proteinuria was reduced by the ASC and MV treatments but not by the EX treatment (Figure 3(d)). Stenotic animals presented a reduction in the levels of urinary Na^+^ excretion compared to sham animals (Figure 3(e)). The ASCs and EXs were able to elevate the urinary Na^+^ levels, but this natriuretic effect was not observed with the MVs. Stenotic animals showed significant urinary K^+^ loss, which was corrected by all the treatments (Figure 3(f)).

### 3.6. ASC and EV Treatments Resulted in Different Gene Expression Responses

**Figure 4:**
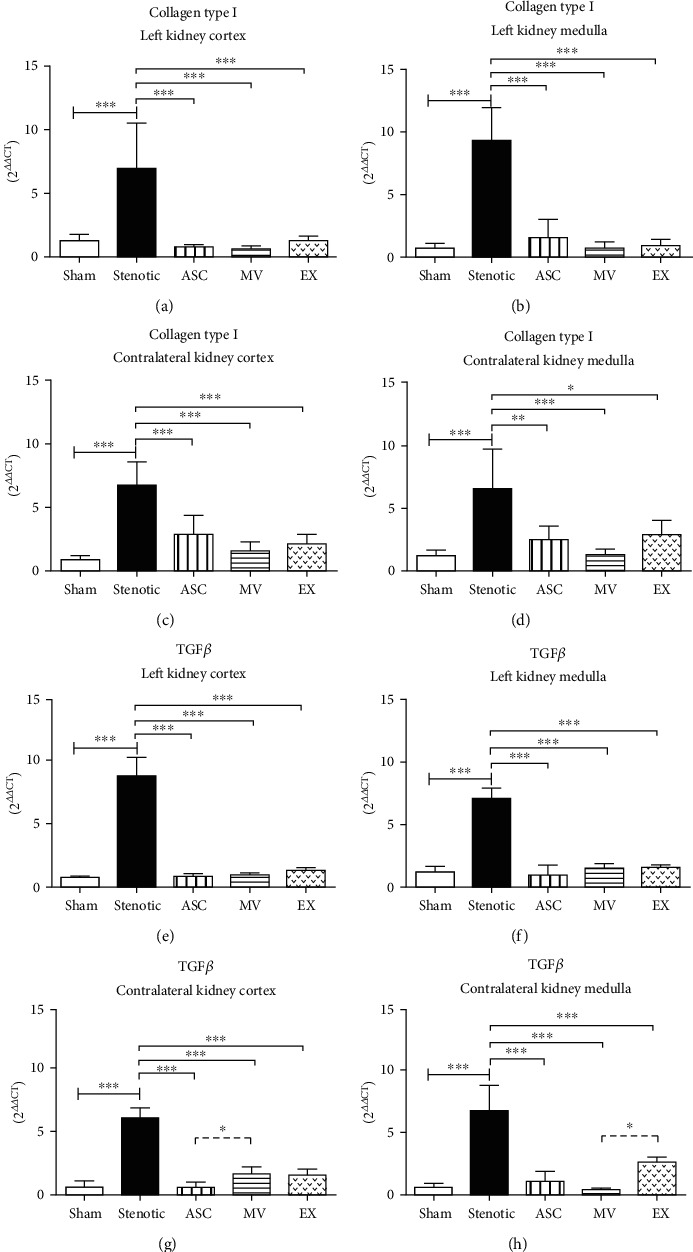
Expression of collagen type I and TGF*β* mRNAs determined by quantitative RT-PCR. Collagen mRNA: stenotic kidney cortex (a), stenotic kidney medulla (b), contralateral kidney cortex (c), contralateral kidney medulla (d). TGF*β* mRNA: stenotic kidney cortex (e), stenotic kidney medulla (f), contralateral kidney cortex (g), contralateral kidney medulla (h). Groups: sham (*n* = 5), stenotic (*n* = 5), stenotic+ASC (*n* = 5), stenotic+MV (*n* = 5), and stenotic+EX (*n* = 5). ^∗^*p* < 0.05, ^∗∗^*p* < 0.01, and ^∗∗∗^*p* < 0.001 (one-way ANOVA followed by Tukey's posttest).

The expression of collagen type I (Col I) was increased in both kidneys of hypertensive animals (Figure 4). The overexpression of collagen I in both kidneys was reduced by all the treatments (Figures 4(a)–4(d)). Similar to collagen expression, TGF*β* gene expression was increased in both kidneys of stenotic animals (Figures 4(e)–4(h)). The ASCs, MVs, and EXs effectively reduced the expression of TGF*β* in the stenotic kidney (Figures 4(e) and 4(f)) and in the contralateral kidney cortex (Figures 4(g) and 4(h)).

**Figure 5:**
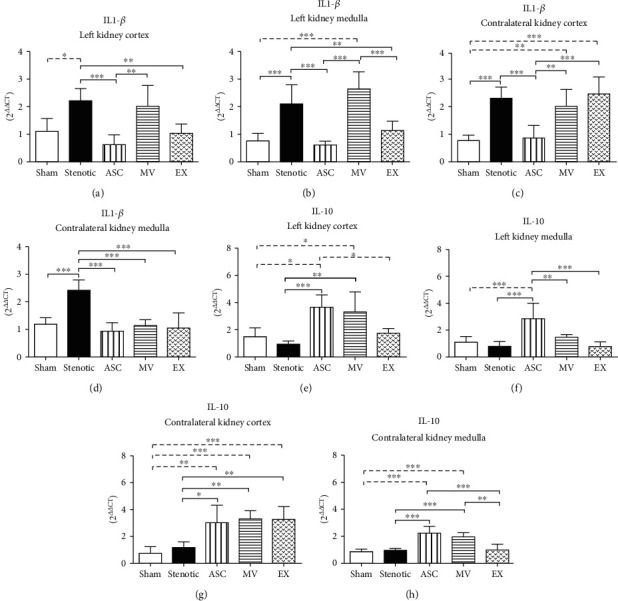
mRNA expression of the proinflammatory cytokine IL-1*β* and the anti-inflammatory cytokine IL-10 determined by quantitative RT-PCR. IL-1*β*: stenotic kidney cortex (a), stenotic kidney medulla (b), contralateral kidney cortex (c), contralateral kidney medulla (d). IL-10: stenotic kidney cortex (e), stenotic kidney medulla (f), contralateral kidney cortex (g), contralateral kidney medulla (h). Groups: Sham (*n* = 5), Stenotic (*n* = 5), Stenotic+ASC (*n* = 5), Stenotic+MV (*n* = 5), and Stenotic+EX (*n* = 5). ^∗^*p* < 0.05, ^∗∗^*p* < 0.01, and ^∗∗∗^*p* < 0.001 (one-way ANOVA followed by Tukey's posttest).

**Figure 6:**
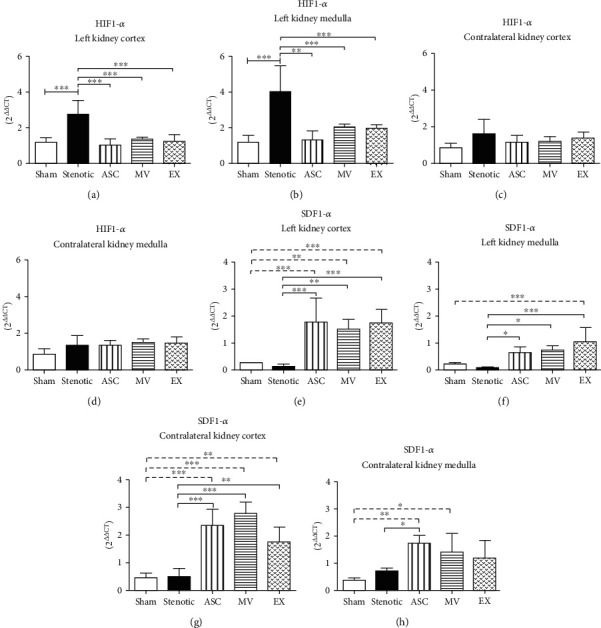
Expression of HIF mRNA determined by quantitative RT-PCR. Stenotic kidney cortex (a), stenotic kidney medulla (b), contralateral kidney cortex (c), contralateral kidney medulla (d). Expression of SDF1-*α* mRNA determined by quantitative RT-PCR. Stenotic kidney cortex (e), stenotic kidney medulla (f), contralateral kidney cortex (g), contralateral kidney medulla (h). Groups: Sham (*n* = 5), Stenotic (*n* = 5), Stenotic+ASC (*n* = 5), Stenotic+MV (*n* = 5), and Stenotic+EX (*n* = 5). ^∗^*p* < 0.05, ^∗∗^*p* < 0.01, and ^∗∗∗^*p* < 0.001 (one-way ANOVA followed by Tukey's posttest).

There was an increase in the expression of the proinflammatory cytokine IL-1*β* in both kidneys of hypertensive animals (cortex and medulla) compared to sham animals (Figures 5(a)–5(d)). The expression of IL-1*β* was effectively reduced in both kidneys, mainly by the ASC treatment (Figures 5(a)–5(d)). In the stenotic kidneys, treatment with ASCs and EXs, but not with MVs, resulted in an improvement in the expression of the proinflammatory cytokine IL-1*β* in both the cortex and medulla (Figures 5(a) and 6(b)). The effects of the treatments were less consistent in the contralateral kidney. In the cortex, only ASCs was able to decrease IL-1*β*, whereas MVs and EXs had no effect. In contrast, all the treatments were effective in the medulla (Figures 5(c) and 5(d)). The induction of renal artery stenosis did not significantly modify the expression of the anti-inflammatory cytokine IL-10 in any of the kidneys (Figures 5(e)–5(h)). Despite this finding, the ASC treatment was effective in increasing the IL-10 expression levels in both kidneys. On the other hand, MVs and EXs were less effective in both kidneys. The MV treatment was effective in increasing IL-10 expression in the stenotic kidney cortex and contralateral kidney (Figures 5(e)–5(h)), while the EX treatment was effective in increasing the expression of this marker only in the contralateral kidney cortex (Figure 5(g)).

As expected, the expression of the hypoxia marker HIF1-*α* was increased in the cortex and medulla of the stenotic kidney (Figures 6(a) and 6(b)) but not in the contralateral kidney (Figures 6(c) and 6(d)). Treatments with ASCs, MVs, and EXs were effective in reducing this marker, and its expression reached values similar to those of the sham group (Figures 6(a) and 6(b)). Animals treated with ASCs showed a significant increase in the expression of the homing stem cell marker SDF1-*α* in the cortex and medulla in both the stenotic and contralateral kidneys (Figures 6(e)–6(h)). Interestingly, the MV- and EX-treated groups also showed an increase in the expression of SDF1-*α* in both kidneys, suggesting that this mRNA can be transported via extracellular vesicles.

## 4. Discussion

In this study, we compared the beneficial effects of whole adipocyte-derived stem cells (ASCs) and 2 distinct populations of secreted EVs (MVs and EXs) in a model of chronic renal hypoxia induced by partial stenosis of the renal artery. In addition to arterial hypertension, renal artery stenosis causes chronic hypoxia, resulting in irreversible renal tissue injury and leading to progressive deterioration of renal function [1], which in turn contributes to worsened hypertension.

We confirmed previous results showing that partial clamping of the left renal artery was effective in producing severe RH, resulting in inflammation and tissue fibrosis in both the clipped and contralateral kidneys [10]. Additionally, hypertensive animals presented lower body weight, cardiac hypertrophy as a consequence of arterial hypertension, reduced stenotic kidney weight, and contralateral kidney hypertrophy as a consequence of hyperflow and hyperfiltration. These results are similar to those observed previously and are characteristics of the model of RH and chronic renal hypoxia [10, 11].

Previous studies [10, 24, 25] demonstrated the beneficial effects of BMSCs on the improvement of renal parenchyma and kidney function in similar animal models of RH [10, 11, 26]. Here, we showed that ASCs induced beneficial effects similar to those induced by BMSCs, with the advantage of being obtained from a more accessible and efficient source than bone marrow.

On the other hand, the benefits of MSCs of different origins have been attributed to their EVs [27], which possess significant potential as a novel alternative to whole-cell therapies [28, 29]. However, it has been demonstrated that different populations of EVs have different contents with distinct biological and regenerative effects [21, 30] and are capable of modulating cellular pathways in recipient cells by different mechanisms, such as direct stimulation, proteins, RNA, miRNA transfer, or surface receptor interaction [31, 32]. The differential ultracentrifugation method used to isolate the EVs in the current study resulted in vesicles that were compatible with EX- and MV-enriched populations; thus, it was possible to compare the regenerative capacity of whole ASCs with that of their distinct EV populations.

ASC treatment resulted in a discrete reduction in SBP; however, compared with that in the untreated hypertensive group, the progressive increase in SBP was blunted in the ASC-treated group, reinforcing the beneficial effects of MSCs of different origins. Interestingly, MVs and EXs produced similar effects on the change in SBP, suggesting that the effects of ASCs on SBP can be mediated by their EVs. Despite the beneficial effects of these treatments in stabilizing SBP, as expected, none of the treatments changed body and organ weight, since both arterial stenosis and hypertension persisted.

It has been shown that EVs derived from MSCs can improve the overall kidney function of CKD patients by increasing the IL-10 levels and repairing the glomerular filtration rate [33]; however, in this study, hypertensive animals showed no alterations in serum creatinine, suggesting a compensatory mechanism of the contralateral kidney and corroborating previously published studies [10, 11, 34]. Despite the lack of change in serum creatinine, signs of functional deterioration, such as significant proteinuria, were observed in the untreated rats. As previously observed with BMSCs [10], the present study showed that ASCs were effective in reducing proteinuria; however, their EVs showed a distinct pattern of protection, since the reduction in proteinuria was also observed with the MV treatment but not with the EX treatment. In contrast, the reduced excretion of sodium observed in hypertensive animals was corrected by ASCs and EXs, but not by MVs. Thus, while ASCs effectively improved all these renal functional parameters, MVs were beneficial in reducing proteinuria and EXs were beneficial in improving Na^+^ excretion. These findings can be explained by the diverse bioactive cargo carried by distinct EV populations [35]. It is well known that EX and MV bear distinct biogenesis pathways, since EX are structures formed from multivesicular bodies in the intracellular compartment, whereas MV are released from the plasma membrane. Thus, differences in the content of distinct EV populations may result in distinct responses. Taken together, these results suggest that the choice of ASCs and/or their specific EV populations for the treatment of RH would be dependent on the parameter that needs to be corrected.

The beneficial effects of ASCs on the renal function parameters, particularly proteinuria and natriuresis, were also previously observed with BMSCs, and these benefits were attributed to reduced inflammation and improved tissue perfusion by neoangiogenesis [11]. On the other hand, the contralateral kidney is the main kidney responsible for sodium excretion in this model of RH, and we have recently shown that treatment with BMSCs improved the capacity of the contralateral kidney to excrete sodium, contributing to the mitigation of volume-dependent hypertension [36]. Despite the compensatory increase in the renal function of the contralateral kidney and its ability to excrete sodium, the contralateral kidney exhibits characteristics of a hypertensive kidney, including inflammation and fibrosis [11]. The cargo of EVs derived from MSCs is associated with many biological functions, such as the regulation of inflammation, the cell cycle, and cell migration [37, 38]; indeed, we observed that all the treatments were equally effective in reducing the expression of the fibrosis markers collagen 1 and TGF*β*. In contrast, the treatments were not equally effective in reducing the inflammatory cytokine IL-1*β* or in improving the expression of the anti-inflammatory cytokine IL-10. Although all the treatments elevated the expression of the anti-inflammatory IL-10, ASCs were the most effective. We believe that these differences can be attributed to the cargo released by each extracellular vesicle type and/or to the ability of these vesicles to reach the proper site in the damaged tissue, but further studies are required to answer these questions.

As expected, the chronic hypoxia in the stenotic kidneys caused an elevation in the expression of hypoxia induction factor-1*α* (HIF-1*α*), and this expression was reduced in the ASC-, MV-, and EX-treated groups. It was previously demonstrated that MSCs were able to induce neoangiogenesis in the stenotic kidneys that could improve renal perfusion and thus reduce HIF1-*α* synthesis. In the present study, we showed that in addition to ASCs, MVs and EXs were also able to suppress HIF1-*α* expression. Taken together, these results suggest that ASCs and both populations of EVs were able to induce neoangiogenesis, improve renal perfusion, and thus reduce HIF1*α* expression.

It has been shown that stem cells have the capacity to migrate to injured tissues in response to chemoattractant factors [39, 40]. The activation of HIF1-*α* can contribute to the recruitment of MSCs, and this may be a key mechanism for the recruitment and migration of MSCs [41]. One piece of evidence that the ASCs were attracted to the injured tissue was the increase in the synthesis of stromal cell-derived factor 1 (SDF-1), which, in turn, played an important role in stem cell homing.

SDF-1*α* can be induced under many physiopathological conditions, including hypoxic and angiogenic environments [42], and can lead to a variety of biological effects, including the homing of cells to the kidney after ischemic injury [43]. The presence of ASCs in both kidneys was verified, indicating that the chemoattraction of these cells to the contralateral kidney was not induced by HIF1-*α* but probably by the presence of high levels of inflammatory molecules in the hypertensive kidney [11, 44].

In the present study, we demonstrated that the highest percentage of ASCs was retained in the stenotic kidney in the first 72 hours, but after 15 days, the cells were still observed in the stenotic kidney and in the contralateral kidney. This fact can be explained by the longer period required for the contralateral kidney to exhibit damage due to severe hypertension. Interestingly, treatment with MVs and EXs also resulted in the upregulation of SDF-1*α* in the kidneys, suggesting that this molecule can be transported through EVs.

## 5. Conclusions

ASCs were as effective as stem cells derived from bone marrow in minimizing the renal effects of renovascular hypertension and chronic renal hypoxia. The EVs released by ASCs exerted beneficial effects similar to those produced by ASCs but with less efficacy in correcting some of the parameters analyzed. The results suggest that differences in the contents of EVs may define their therapeutic roles, and considering the advantages of these vesicles compared with ASCs, the results suggest that the choice of therapeutic strategy should be evaluated according to the parameters to be corrected.

## Data Availability

The datasets generated during and/or analyzed during the current study are available from the corresponding author on reasonable request.

## Abbreviations

RH: Renovascular hypertension
MSCs: Mesenchymal stem cells
ASCs: Adipose-derived stem cell
BMSCs: Bone marrow stem cells
2K-1C: 2 kidneys, 1 clip model
EVs: Extracellular vesicles
MVs: Microvesicles
EXs: Exosomes
IP: Intraperitoneal
SBP: Systolic blood pressure
PBS: Phosphate-buffered saline
FBS: Fetal bovine serum
BW: Body weight
HIF-1*α*: Hypoxia induction factor-1*α*
SDF-1: Stromal cell-derived factor 1
IL-10: Interleukin 10
IL-1*β*: Interleukin 1 beta.

## Supplementary Materials

Supplementary Materials Supplementary Figure 1: mesenchymal stem cell characterization. Representative image of a control culture of Wistar rat adipose tissue (ASCs) (5x magnification) (A). Representative image of calcium deposition in the extracellular matrix stained with Alizarin Red S after 21 days of osteogenic differentiation (5x magnification) (B). Representative image of lipid droplets stained with oil red after 14 days of adipogenic differentiation (5x magnification) (C). Immunophenotyping characterization of ASCs in the third passage (D). Supplementary Figure 2: characterization of microvesicles and exosomes derived from ASCs. Analysis of cell survival at different times (*n* = 4) (A), cell viability (*n* = 4) (B), nanoparticle quantification by Nanosight (*n* = 3) (C), and characterization of microvesicles and exosomes by Western blotting (*n* = 3) (D). Data are presented as the mean ± SD. ^∗∗^*p* < 0.01 vs. MV (one-way ANOVA followed by Tukey's posttest, Student's *t*-test). Supplementary Figure 3: administration and tracking of ASCs labeled with QTracker® and injected intravenously at different times. Representative images of ASCs stained with fluorescent markers and analyzed by flow cytometry from the following tissues from stenotic animals: cortex and medulla of the stenotic kidney (A) and cortex and medulla of the unclipped kidney (B), lung, and heart (C). Percentage of cells stained from the cortex and medulla of the stenotic and unclipped kidney, lung, and heart as determined by flow cytometry (*n* = 2) (D).
