# Supplementary material for: Comparison of the Effects of Mesenchymal Stem Cells with Their Extracellular Vesicles on the Treatment of Kidney Damage Induced by Chronic Renal Artery Stenosis
Source: Stem Cells Int. 2020 Oct 8;2020:8814574. doi: 10.1155/2020/8814574 (PMC7568167; doi:10.1155/2020/8814574)
Supplement: Supplementary Materials — Supplementary Figure 1: mesenchymal stem cell characterization. Representative image of a control culture of Wistar rat adipose tissue (ASCs) (5x magnification) (A). Representative image of calcium deposition in the extracellular matrix stained with Alizarin Red S after 21 days of osteogenic differentiation (5x magnification) (B). Representative image of lipid droplets stained with oil red after 14 days of adipogenic differentiation (5x magnification) (C). Immunophenotyping characterization of ASCs in the third passage (D). Supplementary Figure 2: characterization of microvesicles and exosomes derived from ASCs. Analysis of cell survival at different times (n = 4) (A), cell viability (n = 4) (B), nanoparticle quantification by Nanosight (n = 3) (C), and characterization of microvesicles and exosomes by Western blotting (n = 3) (D). Data are presented as the mean ± SD. ∗∗p < 0.01 vs. MV (one-way ANOVA followed by Tukey's posttest, Student's t-test). Supplementary Figure 3: administration and tracking of ASCs labeled with QTracker® and injected intravenously at different times. Representative images of ASCs stained with fluorescent markers and analyzed by flow cytometry from the following tissues from stenotic animals: cortex and medulla of the stenotic kidney (A) and cortex and medulla of the unclipped kidney (B), lung, and heart (C). Percentage of cells stained from the cortex and medulla of the stenotic and unclipped kidney, lung, and heart as determined by flow cytometry (n = 2) (D). [file 8814574.f1.pdf]

Supplementary Figures

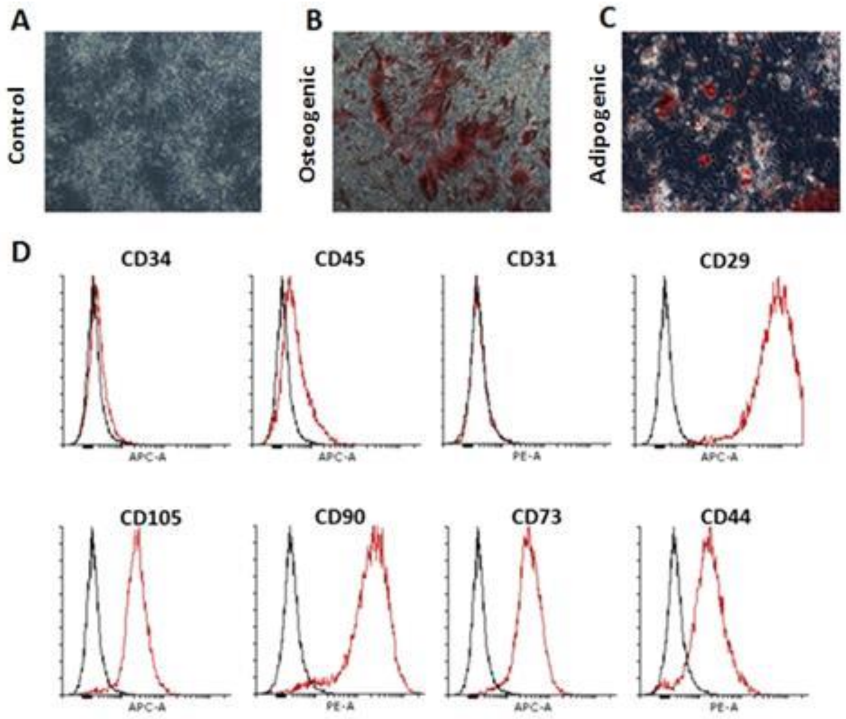

Supplementary Figure 1: Mesenchymal stem cell characterization. Representative image of a control culture of Wistar rat adipose tissue (ASCs) (5x magnification) (A) Representative image of calcium deposition in the extracellular matrix stained with Alizarin Red S after 21 days of osteogenic differentiation (5x magnification) (B). Representative image of lipid droplets stained with oil red after 14 days of adipogenic differentiation (5x magnification) (C) Immunophenotypic characterization of ASCs in the third passage (D).

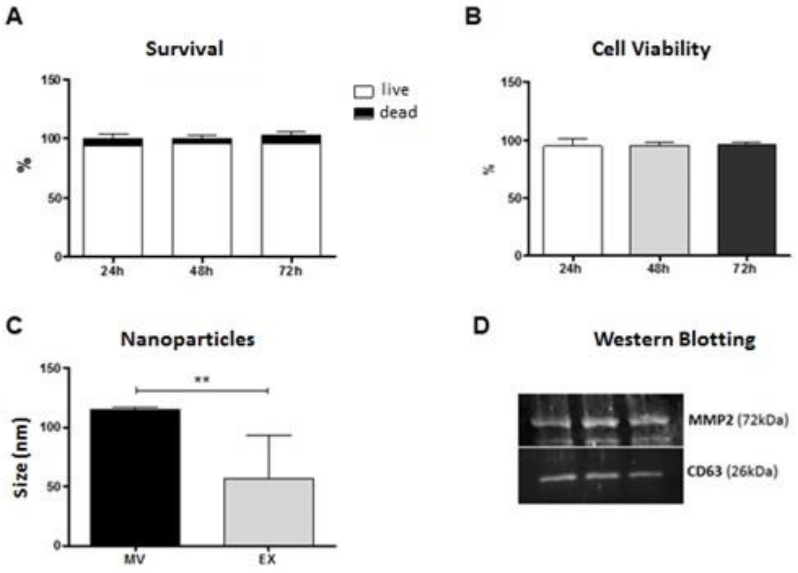

Supplementary Figure 2: Characterization of microvesicles and exosomes derived from ASCs. Analysis of cell survival at different times (n=4) (A) cell viability (n=4) (B) nanoparticle quantification by Nanosight (n=3) (C) characterization of microvesicles and exosomes by Western blotting (n=3) (D) Data are presented as the mean ± SD. ** $p < 0.01$ vs MV (one-way ANOVA followed by Tukey's posttest, Student's t-test).

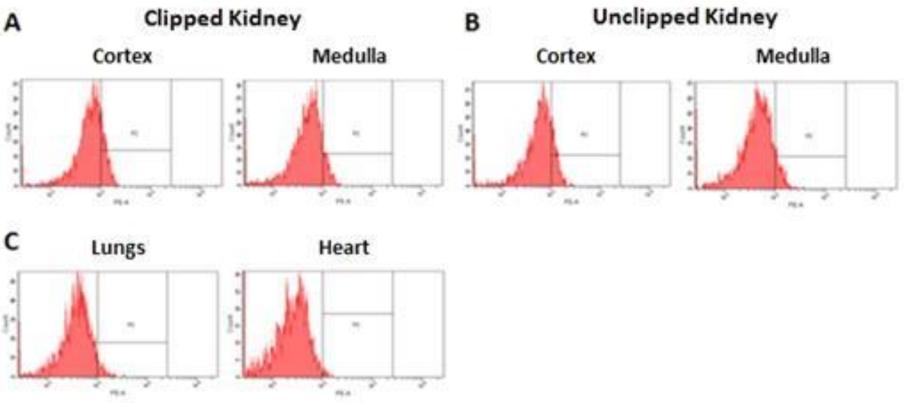

D

ASC (%)					
Tissue	24 hours (%)	48 hours (%)	72 hours (%)	15 days (%)	3rd and 5th week
Cortex of clipped kidney	12,87	21,30	10,23	12,70	16,00
Medulla of clipped kidney	3,13	7,33	1,13	2,55	6,35
Cortex of unclipped kidney	13,50	19,87	8,53	15,80	12,25
Medulla of unclipped kidney	2,57	8,50	1,53	3,45	4,50
Heart	0,77	0,53	0,30	0,90	0,35
Lungs	0,90	4,70	1,23	0,05	0,55

Supplementary Figure 3: Administration and tracking of ASCs labeled with QTracker® and injected intravenously at different times. Representative images of ASCs stained with fluorescent markers and analyzed by flow cytometry from the following tissues from stenotic animals: cortex and medulla of the stenotic kidney (A) cortex and medulla of the unclipped kidney (B), lung and heart (C). Percentage of cells stained from the cortex and medulla of the stenotic and unclipped kidney, lung and heart as determined by flow cytometry (n=2) (D).
